# Multi-Frequency Band Pyroelectric Sensors

**DOI:** 10.3390/s141222180

**Published:** 2014-11-25

**Authors:** Chun-Ching Hsiao, Sheng-Yi Liu

**Affiliations:** Department of Mechanical Design Engineering, National Formosa University, No. 64, Wunhua Rd., Huwei Township, Yunlin County 632, Taiwan; E-Mail: leouishen@gmail.com

**Keywords:** pyroelectricity, sensor, multi-frequency, zinc oxide, thin film

## Abstract

A methodology is proposed for designing a multi-frequency band pyroelectric sensor which can detect subjects with various frequencies or velocities. A structure with dual pyroelectric layers, consisting of a thinner sputtered ZnO layer and a thicker aerosol ZnO layer, proved helpful in the development of the proposed sensor. The thinner sputtered ZnO layer with a small thermal capacity and a rapid response accomplishes a high-frequency sensing task, while the thicker aerosol ZnO layer with a large thermal capacity and a tardy response is responsible for low-frequency sensing tasks. A multi-frequency band pyroelectric sensor is successfully designed, analyzed and fabricated in the present study. The range of the multi-frequency sensing can be estimated by means of the proposed design and analysis to match the thicknesses of the sputtered and the aerosol ZnO layers. The fabricated multi-frequency band pyroelectric sensor with a 1 μm thick sputtered ZnO layer and a 20 μm thick aerosol ZnO layer can sense a frequency band from 4000 to 40,000 Hz without tardy response and low voltage responsivity.

## 1. Introduction

Pyroelectricity, which is electric current generation from time-dependent temperature fluctuations, is useful in many applications, such as pollution monitoring, hot image detectors, intruder alarms, gas analysis and temperature sensors. A pyroelectric material exhibits a spontaneous polarization in the absence of an electric field. Thin-film pyroelectric sensors have many advantages, such as facile integration with on-chip circuitry, uncooled detection, room-temperature operation, speed, lower system costs, portability and a wide spectral response with high sensitivity [1–3]. Pyroelectric sensors have a pyroelectric layer sandwiched between top and bottom electrodes, which are built on thermally isolated structures or substrates to reduce heat loss. The principle of thin-film pyroelectric sensors is based on the pyroelectric effect, namely converting the heat transfer rates to the corresponding electrical signal. The pyroelectric effect is the property of selected dielectric materials with polar point symmetry, which show a spontaneous electrical polarization as a function of temperature. A change in the temperature of the material with respect to time (thermal fluctuations) causes a correspondent variation in the induced charge, thereby producing a pyroelectric current. The dynamic pyroelectric response current (*i_p_*) of pyroelectric sensors can be described by the expression [1]:

(1) $$i_{p}=\eta \times P\times A\times dT/dt$$

where *η* is the absorption coefficient of radiation, *P* is the pyroelectric coefficient of the pyroelectric film, *A* is the electrode area and *dT*/*dt* is the temperature variation rate of the pyroelectric film. When the materials and dimensions of the pyroelectric layers are defined, the absorption coefficient, the pyroelectric coefficient and the electrode area cannot be altered. However, the temperature variation rate has a huge maneuverability according to the design of the patterns, trenches, cavities and structures in the pyroelectric materials.

ZnO is a wide-band gap semiconductor of the II-VI semiconductor group. This semiconductor has several favorable properties, including good transparency, high electron mobility, wide band gap and strong room-temperature luminescence. These properties are used in emerging applications for transparent electrodes in liquid crystal displays, in energy-saving or heat-protecting windows and in electronics as thin-film transistors and light-emitting diodes. ZnO has a relatively large direct band gap of ∼3.3 eV at room temperature. The advantages associated with a large band gap include higher breakdown voltages, the ability to sustain large electric fields, lower electronic noise, and high-temperature and high-power operation. ZnO films have been synthesized by numerous methods, such as metal organic chemical vapor deposition, molecular beam epitaxy, magnetron sputtering, pulsed laser deposition, atomic layer deposition, spray pyrolysis, filtered cathodic vacuum arc technique, sol-gel process and aerosol deposition. The quality of ZnO films obtained by the above methods depends on the specific growth methods and conditions. Thus, the preferential orientation of ZnO films depends on the growth conditions. The pyroelectricity of ZnO is attributable to non-centrosymmetrical crystals, and so it has a specific polar axis along the direction of spontaneous polarization [1–4]. The most densely packed and thermodynamically favorable growth orientation in a ZnO wurtzite structure is one in which the c-axis is perpendicular to the substrate. ZnO films with the c-axis normal to the substrate are preferred in many applications, such as ZnO pyroelectric devices [3,4] and film bulk acoustic resonators [5]. When ZnO is subjected to temperature variations, its internal polarization will produce an electric field. Therefore, increasing the responsivity of a ZnO pyroelectric sensor depends on increasing the temperature variation rate of the ZnO layer, adopting a ZnO film with a strongly preferred orientation towards the c-axis, and using a high-performance thermal-isolation structure. The pyroelectric effect has been applied to environmental energy-harvesting systems. Pyroelectric energy conversion also offers a novel and direct way to convert time-dependent temperature fluctuations into electricity for micropower generators and low-energy-consumption systems [6,7].

The consideration of both the thermal and the electrical circuits is required in the analysis of pyroelectric devices. A radiation with power *W*(*t*) sinusoidally modulated at a frequency *ω* is incident on the top surface of the pyroelectric element (area *A* and thickness *d*), which has emissivity *η*. The pyroelectric element has a thermal capacity *H* and a thermal conductance to the surroundings *G_T_*; the thermal time constant can then be defined as *τ_T_* = *H*/*G_T_*. The thermal capacity can be defined as *H* = *c′dA*, where *c′* is the volume-specific heat. The electrical signal is further amplified by a high-input-impedance field effect transistor, which is used as an amplifier. *R_G_* is the gate resistor, and then the electrical time constant can be defined as *τ_E_* = *R_G_*(*C_E_* + *C_A_*), where *C_E_* is the capacitance of the pyroelectric element; *C_A_* is the capacitance of the amplifier; and *τ_T_* and *τ_E_* are the fundamental factors which determine the frequency response of the pyroelectric sensors. The voltage responsivity (*R_v_*) can be calculated by the following equation [1]:

(2) $$R_{v}=\frac{R_{G}\eta PA\omega }{G_{T}\sqrt{\left(1+\omega^{2}\tau_{T}^{2}\right)}\sqrt{\left(1+\omega^{2}\tau_{E}^{2}\right)}}$$

At a low frequency (*ω* ≪ *τ_T_*^−1^), R_v_ is proportional to the frequency, and is shown as the following equation:

(3) $$R_{v}=\frac{R_{G}\eta PA\omega }{G_{T}}$$

Equation (3) can easily maximize *R_v_* by minimizing *G_T_* (*i.e.*, by adding a thermal insulation layer between the pyroelectric film and the substrates, adopting a suspended structure fabricated by a bulk micromachining technique using anisotropic silicon etching or by using a substrate with a low thermal conductivity). At a high frequency (*ω* ≫ *τ_T_*^−1^; *ω* ≫ *τ_E_*^−1^), *R_v_* is inversely proportional to the frequency, and is shown as the following equation:

(4) $$R_{v}=\frac{\eta P}{c\prime d(C_{E}+C_{A})\omega }$$

Equation (4) can easily maximize *R_v_* by minimizing the thermal capacity of the pyroelectric element *H* (*i.e.*, decreasing the thickness of the pyroelectric element). Moreover, reducing the pyroelectric element's thickness can retard the decline of the voltage responsivity at a high frequency. The frequency at *τ_T_^−1^* is a watershed to distinguish the ranges of low and high frequencies, and the pyroelectric element's thickness determines the value of the thermal time constant (*τ_T_*) under the decided pyroelectric materials and electrode areas. Therefore, a thicker pyroelectric element increases the thermal time constant, which is suitable as the sensor for a low-frequency range. Unlike the thicker element, a thinner pyroelectric element reduces the thermal time constant, which is suitable as the sensor for a high-frequency range. It is difficult to apply a pyroelectric sensor with a single pyroelectric layer to multi-frequency sensing tasks when the materials and dimensions of the pyroelectric layers are already fixed. In other words, the materials and dimensions of the pyroelectric layers directly determine the thermal time constant. Therefore, in this study, a structure with dual pyroelectric ZnO films was designed for a multi-frequency band pyroelectric sensor. The structure mainly comprised two ZnO pyroelectric layers: a thinner ZnO pyroelectric layer deposited by sputtering and a thicker ZnO pyroelectric layer deposited by aerosol deposition (AD). The thinner ZnO film was deposited by RF sputtering. Sputter deposition is a physical vapor deposition (PVD) method of depositing thin films by sputtering. Sputter deposited films have a composition close to that of the source material. Furthermore, sputtered films typically have a better adhesion on the substrate than evaporated films. The properties of ZnO films are affected by sputtering conditions such as the composition of mixed process gases, working pressure, substrate temperature, radio frequency (RF) power, the gap between the target and substrate, and the post-annealing temperature. Moreover, the thicker ZnO film was grown by the AD. In this technique, ceramic films are prepared by ejecting an aerosol consisting of a mixture of ultra-fine ceramic particles and gas from the nozzle to the substrate without vaporization of materials. Compared with the sputtering, screen-printing or sol–gel methods, the AD provides many advantages for producing films in the range of 1∼100 μm thickness with a high deposition rate, low deposition temperature and low cost. The AD method can achieve fine patterning and fabricate a dense structure by the reduction of crystallite size by fracture or plastic deformation at room temperature [8–10]. Although ZnO film grown at a low temperature is available from the AD process, an annealing treatment is one of the most common methods for reducing the defects and improving the quality of as-grown thin film. Therefore, furnace annealing was adopted to improve the ZnO film quality in the present study. The annealing parameters for furnace annealing treatment included temperature, duration, atmosphere and pressure. In particular, annealing temperature and duration are the most effective factors for improving the film quality [11,12].

When a pyroelectric sensor is subjected to temperature variations, its internal polarization produces an electrical field that induces a voltage responsivity between the top and bottom electrodes. The responsivity is proportional to the temperature variation rates in the pyroelectric layers: no temperature variation in the pyroelectric layers results in no internal polarization change and, thus, no voltage response. Temperature variation rates in pyroelectric layers markedly affect the responsivity of pyroelectric sensors. In the present study, transient temperature fields in multi-frequency band pyroelectric sensors were simulated to probe into temperature variation rates at various pyroelectric layers and estimate the response of the sensors. Then, a multi-frequency band pyroelectric sensor was accomplished by designing a structure with dual pyroelectric layers, analyzing the temperature variation fields at various pyroelectric layers, fabricating the multi-frequency band pyroelectric sensor by MEMS process, measuring the responsivity of the sensors by a measurement system and treating and integrating the signals of the sensors by a LabVIEW system.

## 2. Materials and Methods

### 2.1. Design for the Multi-Frequency Band Pyroelectric Sensor

**Figure 1.**
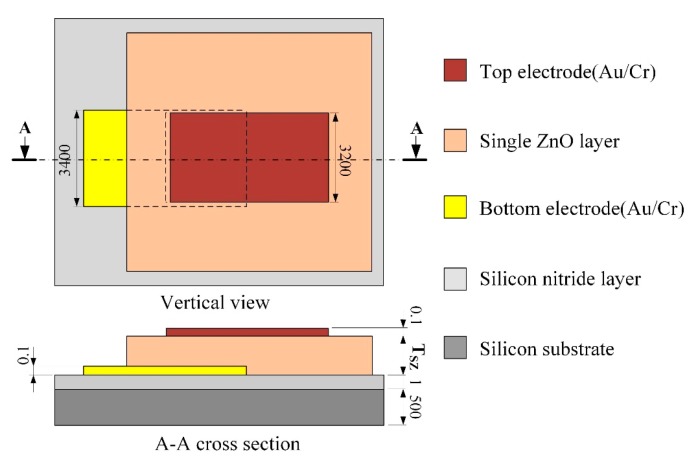
Schematic diagram of a general pyroelectric sensor with a single pyroelectric layer (unit: μm).

A general pyroelectric sensor, as shown in Figure 1, is in a sandwich structure, composed of a single pyroelectric layer sandwiched between top and bottom electrodes, and built on a substrate with an insulating layer for reducing heat and electric loss. As the thickness of the pyroelectric layers determines the thermal time constant (*τ_T_*) under pyroelectric materials and electrode areas already fixed, the thermal time constant determines the optimal working frequency of the pyroelectric sensors. In the present study, a structure with dual pyroelectric film layers was adopted in the design of a pyroelectric sensor with an ability of multi-frequency band sensing; namely, a multi-frequency band pyroelectric sensor.

**Figure 2.**
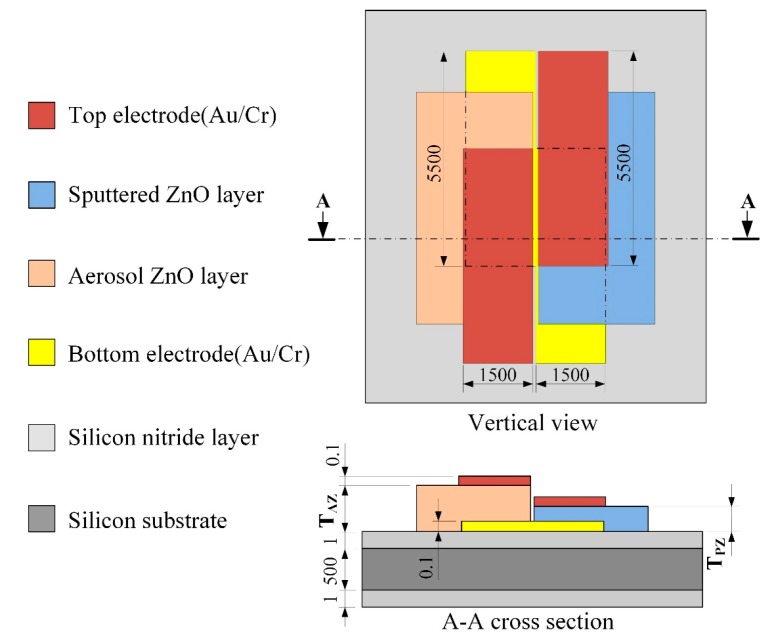
Schematic diagram of the multi-frequency band pyroelectric sensor (unit: μm).

The present multi-frequency band pyroelectric sensor, consisting of a thinner and a thicker ZnO pyroelectric layer and top and bottom electrodes, was built on a silicon substrate with a thermal-insulation (silicon nitride) layer to reduce heat and electric loss. The thinner ZnO pyroelectric layer was named the sputtered ZnO layer, and the thicker ZnO pyroelectric layer was named the aerosol ZnO layer. Figure 2 shows the schematic diagram of the multi-frequency band pyroelectric sensor. The sputtered ZnO layer acted as producer of the responsivity at higher frequency bands, while the aerosol ZnO layer detected the signals of the sensors at lower frequency bands. Sputtering was used to deposit the thinner ZnO films with high quality. Furthermore, the thicker ZnO layer was to detect the signals of the sensors at lower frequency bands, and deposited by the AD. The starting powder was commercially available ZnO (Top Nano Technology Co. Ltd., New Taipei, Taiwan). The properties of the starting ZnO powder are shown in Table 1. Table 2 shows the deposition parameters used for the AD method. The powder was subjected to heat treatment at 150 °C for 1 h using an oven before ZnO films deposited. This could reduce moisture contents in the ZnO powder, and decrease agglomerated particles in ZnO films. Subsequently, furnace annealing was used to the ZnO films to improve the film quality. A furnace annealing system (SJ High Technology Company, Taipei, Taiwan), consisted of a single zone tube furnace with tube furnace model of T21-303, a single zone programmable temperature control console with controller model of SJ-C01, a cooling system and a vacuum system, was adopted for the ZnO film annealing in N_2_ ambient. The parameters applied on furnace annealing of the ZnO films are shown in Table 3. The film thickness was further measured by a surface analyzer (ET-4000AK, Kosaka, Tokyo, Japan).

### 2.2. Simulation

**Figure 3.**
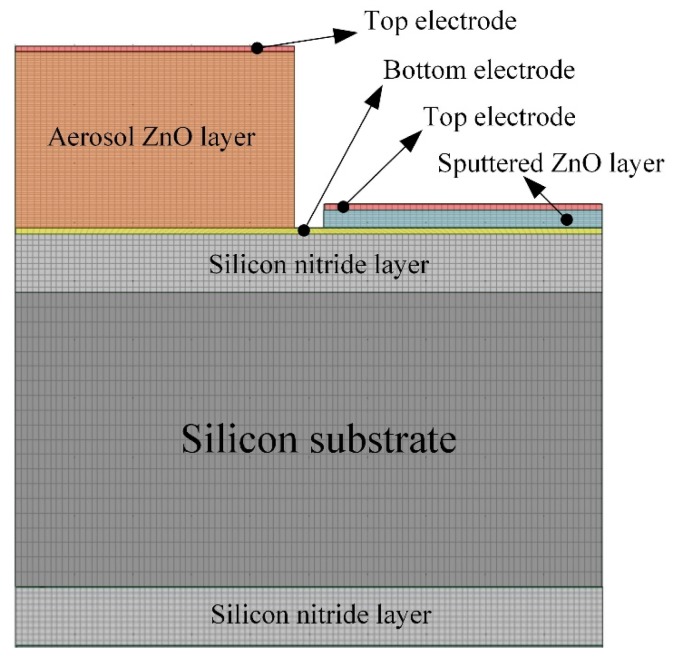
Finite element model for the multi-frequency band ZnO pyroelectric sensor.

Temperature variation rates in the pyroelectric layers markedly affect the responsivity of pyroelectric sensors. Moreover, the temperature variation field is difficult to extract from thin films by experimental measurement. Li *et al.* [13] used a finite element model, built using the commercial software ANSYS, to explore the temperature variation rate in pyroelectric elements with various thermal properties and geometries. Hsiao *et al.* [4] also used a commercial multiphysics software package, COMSOL MULTIPHYSICS^®^, to explore the temperature variation rate in ZnO pyroelectric devices in the design and optimization of a partially covered mesh-type electrode. In the present study, a two-dimensional finite element model was constructed using the commercial multiphysics software COMSOL MULTIPHYSICS^®^ 4.2 to explore the temperature variation rate in the multi-frequency band ZnO pyroelectric sensors. The material parameters of the films and substrate are shown in Table 4. There was an isotropic assumption for the films and substrate properties in this model. The model was meshed using a regular mesh, as shown in Figure 3. The thickness of the sputtered ZnO layer (T_PZ_) was fixed as 0.3 μm, while the thicknesses of the aerosol ZnO layer (T_AZ_) were varied from 1, 2, 3, 4, to 5 μm. The incident irradiation power applied on the top side of the ZnO pyroelectric devices was approximately 1.228 × 10^−12^ W/μm^2^ [13]. The thermal isolation condition was applied to the rear side of the silicon substrate, and the symmetric condition was applied to the two lateral sides as boundary conditions.

### 2.3. Fabrication Process

**Figure 4.**
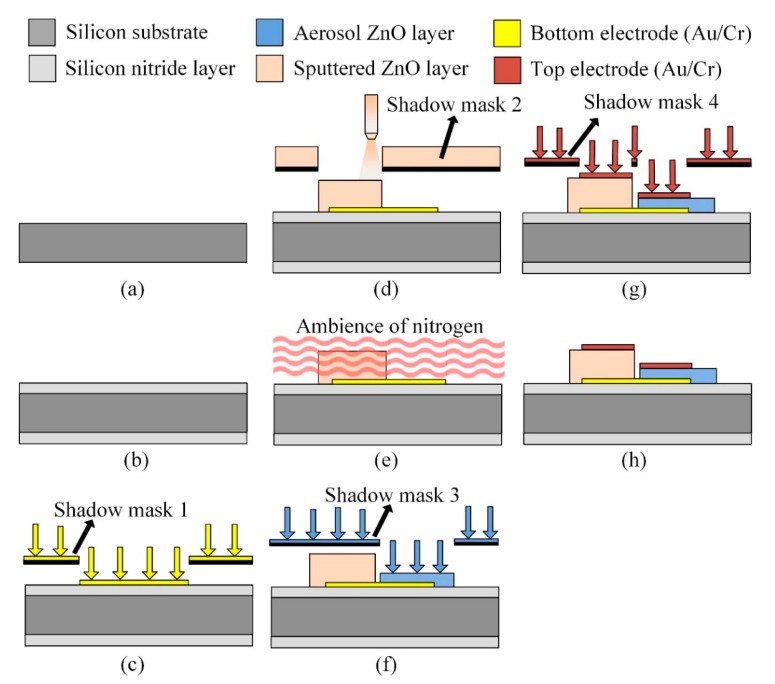
Fabrication flow for the multi-frequency band pyroelectric sensor.

**Figure 5.**
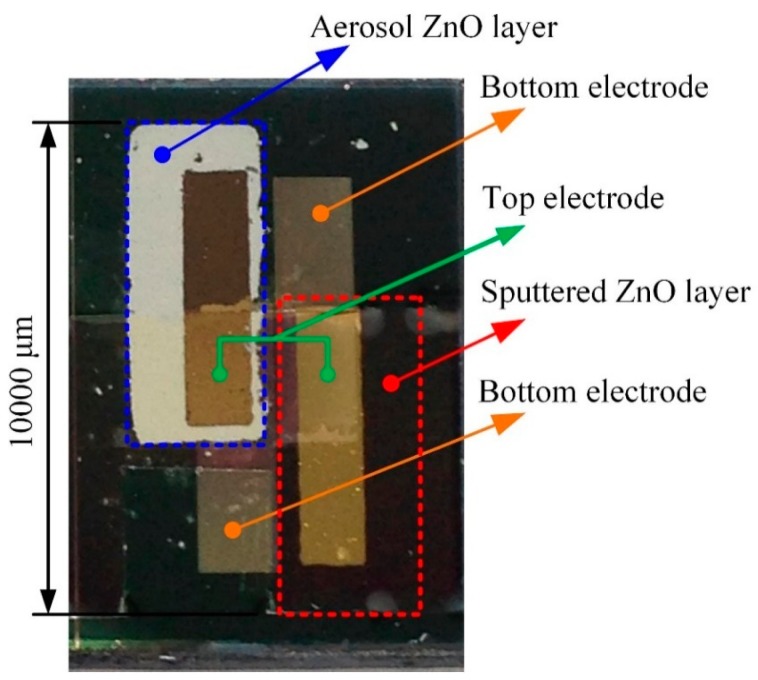
Fabricated multi-frequency band pyroelectric sensor.

The multi-frequency band pyroelectric sensor used both sputtering and AD to extract the advantages of the thin and the thick ZnO pyroelectric film to further integrate the electrical outputs. The process flow of the multi-frequency band ZnO-film pyroelectric sensor was divided into several steps, as follows. A silicon wafer had specifications of (100) p-type, double-side polished, resistivity of 1–10 Ω-cm, and was used as a substrate to support the multi-frequency band pyroelectric sensor, as shown in Figure 4a. Low-stress silicon nitride layers with a thickness of 1 μm were deposited on both sides of the substrate by LPCVD, which was used to reduce or block the heat loss through the silicon substrate, as shown in Figure 4b. The bottom electrode was deposited on the substrate by electron beam (E-beam) evaporation and patterned by the shadow mask method, as shown in Figure 4c. The shadow mask method could simplify process steps and shorten process time. The bottom and top electrode were both composed of gold and chromium. The chromium was an adhesion layer to promote the adhesion between gold and the substrate or ZnO films. The electrodes comprised a 100 nm thick gold layer and a 10 nm thick chromium adhesion layer. The next step was to deposit the aerosol ZnO film with a thickness of about 20 μm by the AD, as shown in Figure 4d, and then those were promoted by furnace annealing, as shown in Figure 4e. Subsequently, a 1 μm thick ZnO layer (the sputtered ZnO layer) was deposited on the bottom electrode by RF magnetron sputter, as shown in Figure 4f. A ZnO target with 99.99% purity was adopted. Prior to the film deposited, the ZnO target was pre-sputtered for 15 min to remove any surface impurity. The chamber was pumped with base pressure up to 8 × 10^−7^ Torr before sputtering. The chamber was then filled with the mixture of argon and oxygen with the gas-mixing ratio of 5:3. The RF power was kept at 120 W. The chamber pressure was 2 mTorr during the film deposited. The substrate was heated up to 200 °C while deposition, which could help to make better ZnO film quality. The top electrode was deposited on the sputtered and the aerosol ZnO film by E-beam and patterned by the shadow mask method, as shown in Figure 4g. Finally, the wet etchant of CH_3_COOH:H_3_PO_4_:H_2_O = 1:1:10 was used to pattern ZnO layers to open the bonding pads of the bottom electrode, as shown in Figure 4h. The fabricated multi-frequency band pyroelectric sensor is shown in Figure 5.

### 2.4. Signal Treatment and Measurement

The multi-frequency band pyroelectric sensor used the advantages of the thinner and the thicker ZnO pyroelectric film to integrate the electrical outputs yielded from those films into an all-round signal. The voltage responsivity of V_P_ was generated from the sputtered ZnO film for shouldering a high-frequency response, and the voltage responsivity of V_A_ was generated from the aerosol ZnO film for taking a low-frequency response. The integrated and treated electrical signal was V_T_.

**Figure 6.**
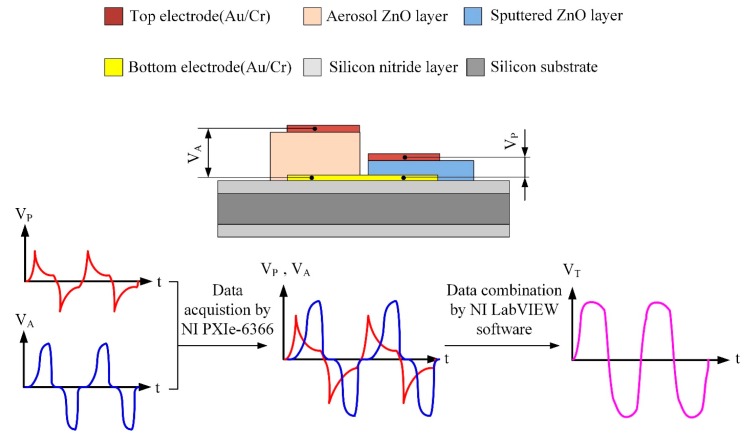
Schematic diagram of signal treatment flow.

**Figure 7.**
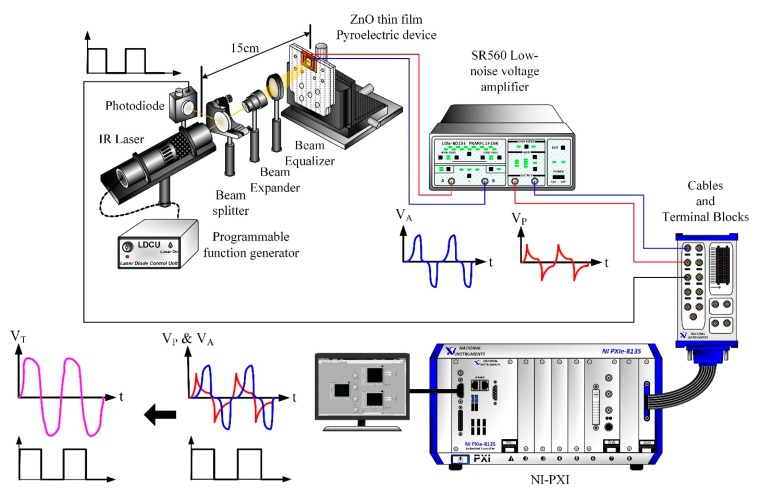
Schematic diagram of voltage responsivity measurement system.

A diagram of the flow of the signal treatment is shown in Figure 6. A voltage responsivity measurement system, as Figure 7, was adopted to appraise the performance of the multi-frequency band ZnO thin-film pyroelectric sensor.

The radiation source was a calibrated infrared (IR) laser of 900 nm wavelength and 7 mW maximum power. The laser beam was molded as a square wave with a modulated frequency (ω) by a programmable function generator. A prism was used to split the modulated beam into two beams, which had the same power: one was reflected on a photodiode as the reference signal, and the other was expanded and equalized via a beam expander and a beam equalizer such that the beam spot could uniformly cover the entire region of the top electrode of the sensors. Both the responsivities of V_P_ and V_A_ were filtered, amplified, modulated and combined as an integrated voltage responsivity by NI LabVIEW software. Moreover, both the integrated voltage responsivity of the sensors and the reference signal of the photodiode were acquired, recorded and displayed using NI LabVIEW system consisted of a case of NI PXIe-1082, a controller of NI PXIe-8135, a data acquisition card of NI PXIe-6366 and NI LabVIEW 2012 software.

## 3. Results and Discussion

**Figure 8.**
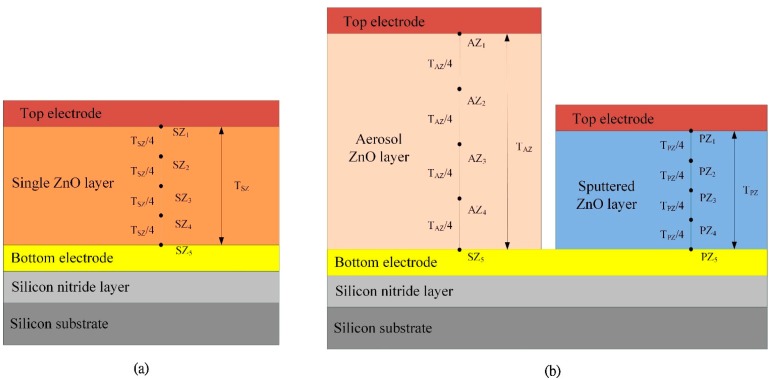
The points defined for explaining the transient temperature fields in the ZnO pyroelectric devices for (**a**) the single layer type and (**b**) the dual layer type.

The responsivities of pyroelectric devices are proportional to the temperature variation rate in the pyroelectric layers. Therefore, the transient temperature fields in multilayer pyroelectric devices with various geometric structures were simulated. Moreover, the response time was estimated. The points, as shown in Figure 8, were used to interpret the temperature variation rates in the ZnO pyroelectric devices with the single pyroelectric layer and the dual pyroelectric layers. Points SZ_1_, SZ_3_ and SZ_5_ were, respectively, located on the top, the middle and the bottom of the single ZnO layer. The thickness of the single ZnO layer was named as T_SZ_. Points PZ_1_, PZ_3_ and PZ_5_ were, respectively, located on the top, the middle and the bottom of the sputtered ZnO layer. The thickness of the sputtered ZnO layer was named as T_PZ_. Points AZ_1_, AZ_3_ and AZ_5_ were, respectively, located on the top, the middle and the bottom of the aerosol ZnO layer. The thickness of the aerosol ZnO layer was named as T_AZ_.

**Figure 9.**
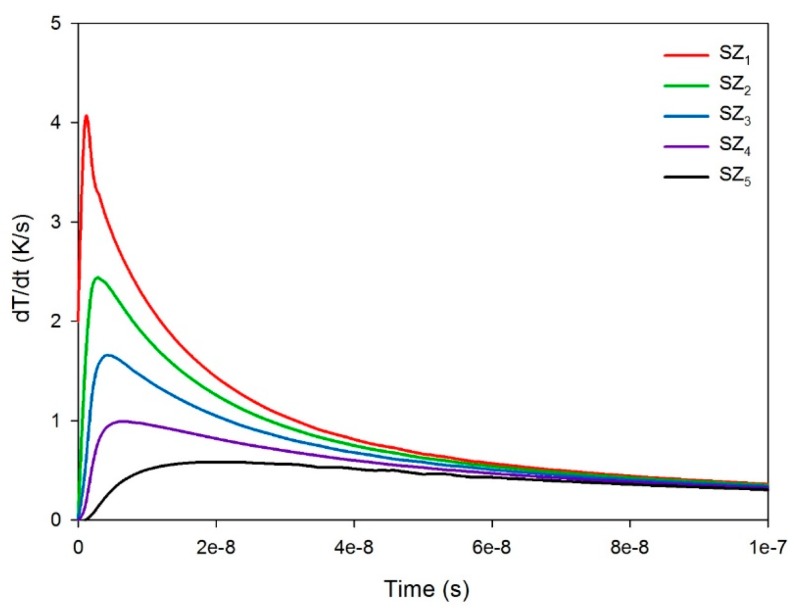
Relationship between the temperature variation rate and time at points SZ_1_ to SZ_5_ under a 0.3 μm thick single ZnO layer.

**Figure 10.**
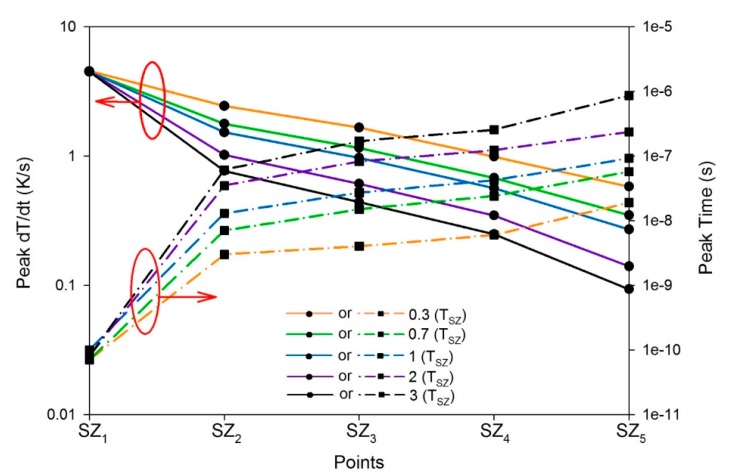
The peak temperature variation rate and the peak time of the maximum temperature variation rate at points SZ_1_ to SZ_5_ under a single ZnO layer 0.3 to 3 μm thick.

For the pyroelectric sensor with the single ZnO layer, Figure 9 shows the relationship between the temperature variation rate and time in the single ZnO layer along the thickness direction from points SZ_1_ to SZ_5_, when a single ZnO layer with a thickness of 0.3 μm was used to fabricate the ZnO pyroelectric sensor. The temperature variation rate increased when the point approached to the top side of the single ZnO layer, because incident radiation was applied on the top electrode. Moreover, the peak of the temperature variation rate moved leftward, and then the peak time of the maximum temperature variation rate also reduced, thus decreasing the response time. Figure 10 shows the peak temperature variation rate and the peak time of the maximum temperature variation rate at points SZ_1_ to SZ_5_, when the thickness of the single ZnO layer varied from 0.3 to 3 μm. A thinner ZnO layer was conducive to enhancing the temperature variation rate and improving the response speed in the pyroelectric sensor. However, the temperature variation rate at various locations in the ZnO film had different values and distributions at different time periods. Using the peak temperature variation rate and the peak time at point SZ_1_ could not discriminate the properties of the pyroelectric sensor with various thicknesses of the ZnO films. Therefore, a conservative response was to adopt the properties at point SZ_5_ for inspecting the sensors, because the peak temperature variation rate and the peak time at point SZ_5_ were disappointing, as compared to points SZ_1_, SZ_2_, SZ_3_, and SZ_4_ under the specific thickness of ZnO film being used. Therefore, improvement in the temperature variation rate at the point SZ_5_ possessed the lowest temperature variation rate certainly enhanced the responsivity of the pyroelectric devices.

**Figure 11.**
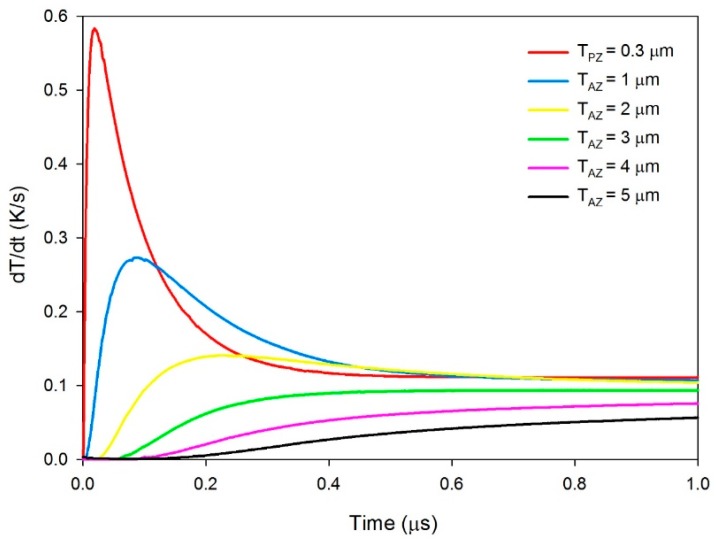
Relationship between the temperature variation rate and time at points PZ_5_ and AZ_5_ in the multi-frequency band pyroelectric sensor under sputtered ZnO film with a constant thickness of 0.3 μm and aerosol ZnO film with various thicknesses from 1 to 5 μm.

For the multi-frequency band pyroelectric sensor, Figure 11 shows the relationship between the temperature variation rate and time at points PZ_5_ and AZ_5_ in the dual ZnO layers, when sputtered ZnO film with a constant thickness of 0.3 μm and aerosol ZnO film with various thicknesses from 1 to 5 μm were used to fabricate the ZnO pyroelectric sensors.

**Figure 12.**
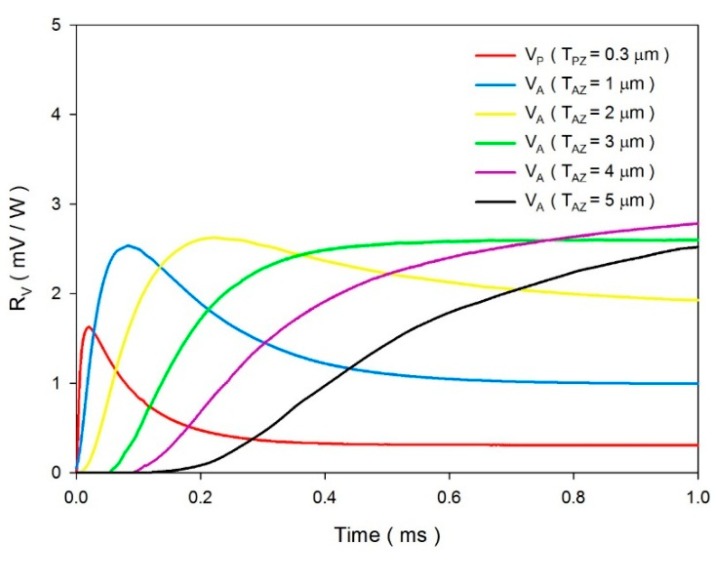
Relationship between the voltage responsivities and time in the multi-frequency band pyroelectric sensor under the sputtered ZnO film with a constant thickness of 0.3 μm and the aerosol ZnO film with various thicknesses from 1 to 5 μm.

**Figure 13.**
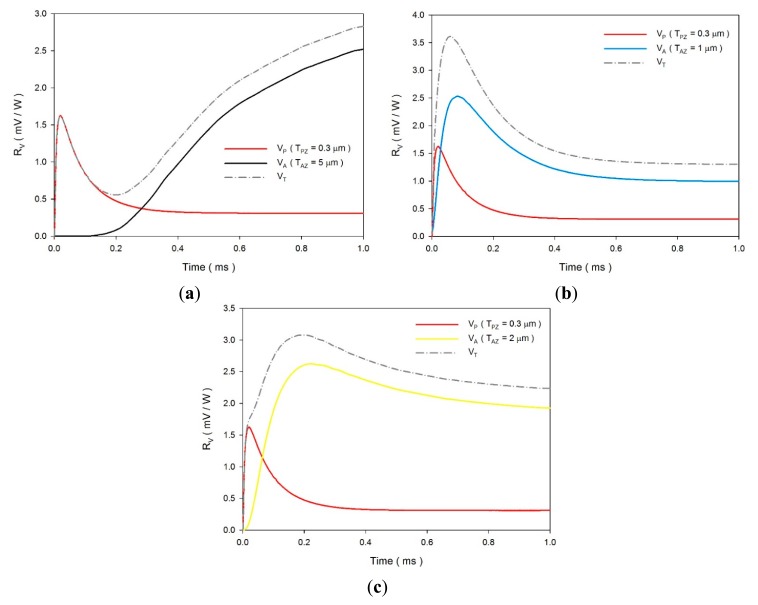
The integrated voltage responsivities (V_T_) generated by combining V_P_, generated by a 0.3 μm thick sputtered ZnO film, with V_A_, generated by various thicknesses of aerosol ZnO film: (**a**) T_AZ_ = 5 μm; (**b**) T_AZ_ = 1 μm; (**c**) T_AZ_ = 2 μm.

The sputtered ZnO film possessed a higher temperature variation rate and a shorter response time, while the aerosol ZnO film possessed a lower temperature variation rate and a longer response time. Increasing the thickness of the ZnO layer reduced the temperature variation rate and increased the peak time of the maximum temperature variation rate. Therefore, a ZnO film with a constant thickness in a pyroelectric device could not handle various multi-frequency sensing tasks. In fact, the pyroelectric devices use the voltage responsivity for presenting the performance of the sensors. Hence, the voltage responsivities were calculated for estimating the electrical outputs of the sensors. The voltage responsivity (*R_v_*) is defined as the signal generated when the pyroelectric sensors are exposed to a modulated radiation. Moreover, when the pyroelectric sensors are connected to a high impedance amplifier, the observed signal is equal to the voltage produced by the charge. *R_v_* can be expressed as [1]:

(5) $$R_{v}=\frac{i_{p}}{Y\times W_{0}}$$

where *i_p_* is the dynamic pyroelectric response current, *W_0_* is the magnitude of the incident radiation and *Y* is the electrical admittance, as:

(6) $$Y=R_{G}^{-1}+i\omega C_{T}$$

where *R_G_* is the gate resistor, *ω* is the modulated frequency of the incident radiation, *C_T_* is the sum of *C_E_* and *C_A_*, *C_E_* is equal to *ε_0_ε_r_ A*/*d*, *ε_0_* is the vacuum permittivity (8.85 × 10^−12^ F/m) and *ε_r_* is the relative permittivity or dielectric constant of the materials. *R_v_* was estimated using the simulated data of the temperature variation rates and Equations (1), (5) and (6). The relative conditions for computing the voltage responsivity are shown in Table 5. Figure 12 shows the relationship between the voltage responsivities and time in the multi-frequency band pyroelectric sensor when sputtered ZnO film with a constant thickness of 0.3 μm and aerosol ZnO film with various thicknesses from 1 to 5 μm were used to fabricate the ZnO pyroelectric sensors. Furthermore, the temperature variation rates at points PZ_5_ and AZ_5_ were used to estimate the voltage responsivities generated by the sputtered and the aerosol ZnO film, respectively. The curve of the voltage responsivity generated by the sputtered ZnO film (V_P_) with a rapid response could be attributed to a thinner ZnO layer with a large temperature variation rate and a small thermal capacity, while that generated by the aerosol ZnO film (V_A_) with a tardy response could be attributed to a thicker ZnO layer with a large thermal capacity and a small temperature variation rate. Therefore, V_P_ was responsible for shouldering a high-frequency response, and V_A_ was responsible for shouldering a low-frequency response. V_A_ was larger than V_P_ at the low-frequency band, which could be attributed to the thicker ZnO layer with a small *C_E_*. The peak time of the maximum temperature variation rate increased when the thickness of the aerosol ZnO film increased due to the small temperature variation rate and large thermal capacity. The curves of V_P_ and V_A_ had an apparent intersection, which could be used for differentiating between the high- and the low-frequency bands. V_T_ was the integrated voltage responsivity by combining V_P_ with V_A_. However, integrating V_P_, generated by a 0.3 μm thick sputtered ZnO film, with V_A_, generated by a thicker aerosol ZnO film, was disadvantageous because the voltage responsivity did not produce a compensatory effect between the peak time of V_P_ and V_A_, as shown in Figure 13a. Furthermore, integrating V_P_, generated by a 0.3 μm thick sputtered ZnO film, with V_A_, generated by a thinner aerosol ZnO film, was also unsuitable because the reinforced sensing frequency band was narrow, as shown in Figure 13b. An optimal V_T_ needed a suitable combination, matching the thicknesses of the sputtered and the aerosol ZnO films. Obviously, as shown in Figure 13c, the voltage responsivity of V_T_ could shoulder the sensing tasks of the pyroelectric devices in a multi-frequency band.

**Figure 14.**
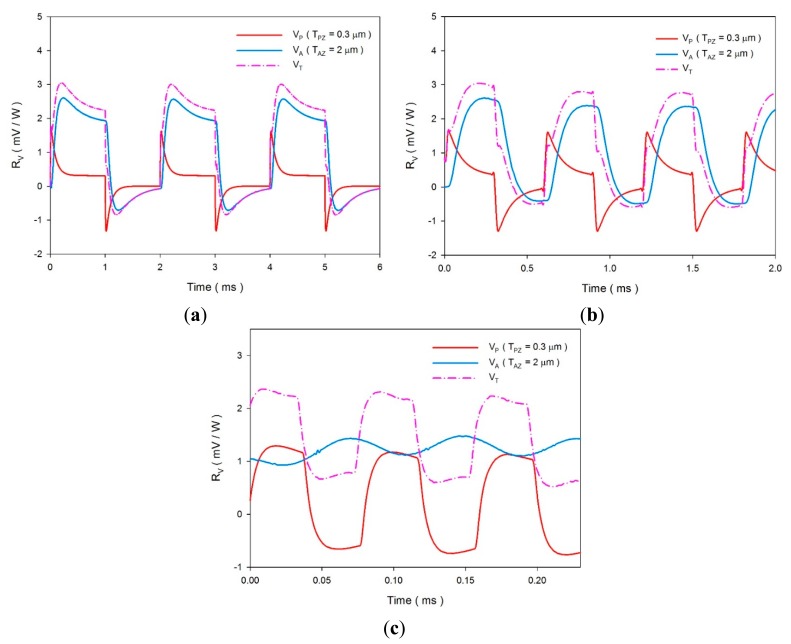
Voltage responsivities of the multi-frequency band pyroelectric device with T_PZ_ = 0.3 μm and T_AZ_ = 2 μm under the incident irradiation power modulated with various chopping frequencies of about (**a**) 10,000 Hz; (**b**) 33,000 Hz and (**c**) 250,000 Hz.

The transient temperature fields with various chopping frequencies were simulated for further calculating voltage responsivities when the multi-frequency band pyroelectric devices were exposed to irradiation power with various chopping periods. A square waveform was produced using a programmable function generator to modulate the irradiation power with a peak value of 1.228 × 10^−12^ W/μm^2^ and chopping frequencies of about 10,000, 33,000 and 250,000 Hz. Figure 14 shows the voltage responsivities of the multi-frequency band pyroelectric device with T_PZ_ = 0.3 μm and T_AZ_ = 2 μm when the incident irradiation power was modulated with various chopping frequencies of about 10,000, 33,000 and 250,000 Hz. The shape and amplitude of the integrated voltage responsivity of V_T_ approached to that of V_P_ generated by the sputtered ZnO film when the chopping frequency was near a high frequency about 250,000 Hz. Moreover, the shape and amplitude of the integrated voltage responsivity of V_T_ approached to that of V_A_ generated by the aerosol ZnO film when the chopping frequency was near a low frequency about 10,000 Hz. V_T_ had a compensatory effect between V_P_ and V_A_. V_T_ redeemed the drawbacks of V_A_ with a tardy response at a high-frequency band and V_P_ with a low voltage responsivity at a low-frequency band. This implied that the integrated voltage responsivity of V_T_ generated by the multi-frequency band pyroelectric devices could present the performances of various pyroelectric devices at various sensing frequency bands.

**Figure 15.**
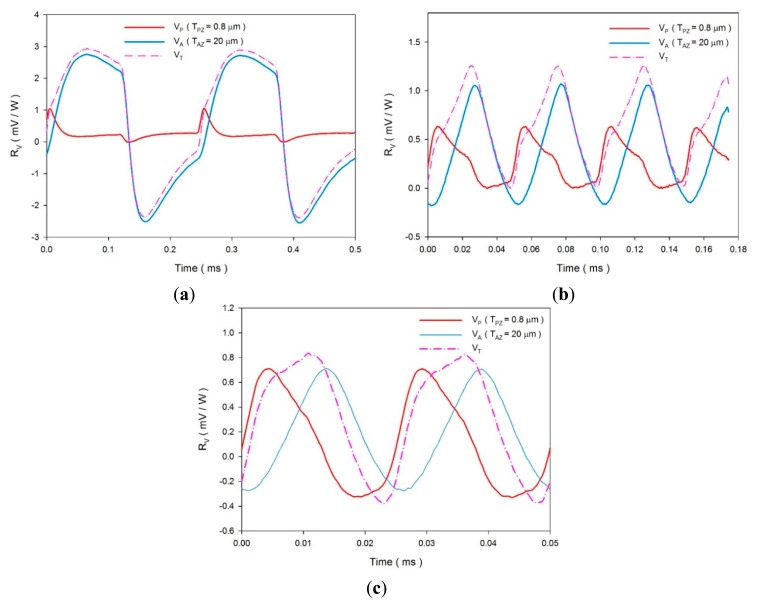
Voltage responsivities of the fabricated multi-frequency band pyroelectric device with TPZ = 1 μm and T_AZ_ = 20 μm under the incident irradiation power modulated with various chopping frequencies of about (**a**) 4000 Hz; (**b**) 20,000 Hz and (**c**) 40,000 Hz.

An experimental setup was used to verify the above analytical results. The IR laser beam was chopped and molded as a square wave with various modulated frequencies (4000, 20,000 and 40,000 Hz) to obtain temperature variation rates in the multi-frequency band pyroelectric device by means of a programmable function generator. Figure 15 shows the voltage responsivities of the fabricated multi-frequency band pyroelectric device with T_PZ_ = 1 μm and T_AZ_ = 20 μm when the incident irradiation power was modulated with various chopping frequencies of about 4000, 20,000 and 40,000 KHz. The fabricated multi-frequency band pyroelectric device at the modulated frequency of 4000 presented a low-frequency property. V_T_ presented a large temperature variation rate, but it did not possess a tardy response. Furthermore, the fabricated multi-frequency band pyroelectric device at the modulated frequency of 40,000 presented a high-frequency property. Although V_T_ could not improve the temperature variation rate, the sensing frequency band increased. This implied that the voltage responsivity could hold on a peak value with a larger sensing frequency band. Hence, the multi-frequency band pyroelectric device could enhance pyroelectric sensors detecting subjects with various speeds or frequencies.

## 4. Conclusions

A pyroelectric sensor with a single pyroelectric layer has only a single sensing frequency band. Multi-frequency sensing tasks could improve applications of pyroelectric sensors for detecting subjects with various speeds or frequencies. A multi-frequency band pyroelectric sensor was successfully designed, analyzed and fabricated in the present study. The combination of a thinner sputtered ZnO layer and a thicker aerosol ZnO layer proved useful in the present design. The ranges of the multi-frequency sensing tasks could be predicted by the proposed design and analysis to match the thicknesses of the sputtered and the aerosol ZnO layers for producing a compensatory effect and extending the reinforced sensing frequency band. The fabricated multi-frequency band pyroelectric sensor with a 1 μm thick sputtered ZnO layer and a 20 μm thick aerosol ZnO layer could cover the sensing frequency band from 4000 to 40,000 Hz without tardy response and low voltage responsivity.

## Figures and Tables

**Table 1.** Material properties and geometry of commercially ZnO powder.

**Item**	**Data**
Appearance	White powder
Density	5.61 g/cm^3^
Specific surface area	17 m^2^/g
Particle form	Sheet
Particle size	300 nm (Diameter) × 20 nm (Thickness)

**Table 2.** Process parameters for ZnO films deposited by the AD method.

**Item**	**Data**
Starting powder	ZnO
Substrate	Silicon
Pressure difference between deposition and aerosol chambers	140 (Torr)
Carrier gas	Nitrogen
Consumption of carrier gas	3 (L/min)
Orifice size of nozzle	0.4 × 10 (mm × mm)
Substrate temperature	25 (°C)
Deposition area	70 × 70 (mm × mm)
Distance between nozzle and substrate	5 (mm)
Scanning rate	10 (mm/s)
Deposition rate	8.2 (nm/s)

**Table 3.** Process parameters for ZnO films treated by furnace annealing.

**Item**	**Data**
Holding temperature	800 (°C)
Duration	15 (min)
Speed for raising temperature	10.4 (°C/min)
Ambience	Nitrogen
Cooling type	Cooling in furnace

**Table 4.** Material parameters used for the simulation.

**Material**	**Thermal Conductivity (Wm^−1^·K^−1^)**	**Specific Heat (Jg^−1^·K^−1^)**	**Density (g·cm^−3^)**	**Thickness (μm)**
Silicon substrate	163	0.703	2.330	5
Silicon nitride	20	0.700	3.100	1
Electrodes	317	0.129	19.300	0.1
sputtered and aerosol ZnO layers	6	0.125	5.676	T_PZ_ and T_AZ_

**Table 5.** Relative conditions for estimating *Rv*.

**R_G_ (MΩ)**	***C****_A_* **(pF)**	***P* (10^−4^ C/m^2^·K)**	***A* (mm^2^)**	***ε****_r_* **(Unit-less)**	***W****_0_* **(W/μm^2^)**
22	6	0.1	9	11	1.228 × 10^−12^
